# 

**Published:** 1842-04

**Authors:** 


### BOOKS RECEIVED FOR REVIEW.

ENGLISH.

1. Reply to Dr. Chalmers's Objections to an Improvement of the Legal Provision for the Poor in Scotland. By W. P. Alison, M.D. &c.—Edin. 1841. 8vo, pp. 56.

2. The Retrospective Address delivered at the Ninth Anniversary Meeting of the Provincial Medical and Surgical Association. By R. J. N. Streeten, M.D.—Worcester, 1841. 8vo, pp. 904.

3. The Double-Flap and Circular Amputations contrasted, being an abstract of a first Prize Essay at the University of Edinburgh. By F. N. Machardy, A.M. & M.D., Surgeon.—London, 1841. 8vo, pp. 53.

4. The means of promoting and preserving Health. By T. Hodgkin, M.D. Second Edition, with Additions.—London, 1841. 8vo, pp. 480.

5. An Investigation of the proposed Scheme of Medical Reform in reference to Chemists and Druggists. By G. Crook, M.P.S.—Lond. 1841. 8vo, pp. 32.

6. The Anatomy of the Arteries of the Human Body. By R. Quain.—Part 10. 12s.

7. The Chemist; or Reporter of Chemical Discoveries and Improvements. Edited by C. Watt, and John Watt, Jun. Vol. ii. —Lond. 1841. 8vo, pp. 392.

8. Notes on the United States of North America, during a Phrenological Visit in 1838-9-40. By George Combe.—Edinb. 1841. 3 vols. 8vo, pp. 371, 400, 488. 31s. 6d.

9. Human Physiology; illustrated by Engravings. By R. Dunglison, M.D., &c. Fourth Edition, with numerous additions and modifications.—Philadelphia, 1842. Two vols. 8vo, pp. 579, 657.

10. Elements of Materia Medica and Pharmacy. By O'Bryen Bellingham, M.D., Lecturer on Mat. Med. Dublin. Edited by Arthur Mitchell, M.D. Part I.—Dublin, 1841. 8vo, pp. 302, 6s.

11. A Collection of Plates illustrating the Theory and Practice of Midwifery, accompanied by an explanatory Text. Translated from the French by Em. Debout, M.D. Part I.—London and Paris, 1841. Large Folio. Plates xxvii.

12. Natural History of Man. By J. C. Prichard, M.D., F.R.S., M.R.I.A. Illustrated with Plates. Nos. I, II, III. 2s. 6d. each. —Lond. 1842. 8vo.

13. Atlas illustrative of the Anatomy of the Human Body. By J. Cruveilhier, M.D. The figures drawn from nature, by Em. Beau, with explanations by C. Bonamy, M.D. Parts I—IV, 2s. 6d. each.—Lond. 1841-2. Royal 8vo.

14. Further Observations on Medical Reform. By J. Kidd, M.D., Regius Professor of Medicine in the University of Oxford.—Lond. 1841-2. 8vo, pp. 16. 1s.

15. An Essay on the influence of constitution in the production of disease. By E. O. Hocken, M.D.—Lon. 1841. 12mo, pp. 64. 2s. 6d.

16. A Treatise on Dislocations and Fractures of the Joints. By Sir A. Cooper, Bart. F.R.S. A new Edition much enlarged. Edited by Bransby B. Cooper, F.R.S.—Lon. 1842. 8vo, pp. 576. 20s.

17. An Exposition of the Pathology of Hysteria: elucidated by a reference to the diagnosis &c. of hysterical Amaurosis. By E. O. Hocken, M.D.—Lond. 1842. 12mo, pp. 32.

18. On the Treatment of Stone in the Bladder by medical and mechanical means. By R. Willis, M.D.—Lon. 1842. 8vo, pp. 103.

19. Spinal and Nervous Diseases, Rheumatism, and Paralysis: or Cases and Observations illustrating an improved treatment. By J. H. Robertson, M.D.—Glasg. 1842. 8vo, pp. 119.

20. Minutes of the Committee of Council of Education: with Appendices. 1840-41.—Lond. 1841. 8vo, pp. 482.

21. Catalogue of the Preparations in the Anatomical Museum of George Langstaff, Surgeon.—Lond. 1842. 8vo, pp. 518. 10s.

22. A practical Treatise on Venereal Diseases; or Critical and Experimental Researches on Inoculation applied to the study of these affections. By Ph. Ricord, M.D. Translated from the French by H. P. Drummond, M.D.—Lond. 1842. 8vo, pp. 384.

23. The Administration of Medical Relief to the poor under the Poor Law Amendment Act, &c.—Lond. 1842. 8vo, pp. 136.

24. Letter to the Earl of Aberdeen on the state of the Schools of Chemistry in the United Kingdom. By W. Gregory, M.D., Professor of Chemistry in the University and King's College, Aberdeen.—Lond. 1842. 8vo, pp. 35.

25. Principles of Surgery. By J. Syme, F.R.S.E., Professor of Clinical Surgery in the University of Edinburgh. Third Edition enlarged.—Lond. 1842. 8vo, pp. 503, with plates and woodcuts. 21s.

26. Hydropathy; or the Cold Water Cure, as practised by Vincent Priessnitz. By R. T. Claridge, Esq.—Lon. 1842. 8vo, pp. 318. 5s.

27. A Practical Treatise on Auscultation. By M. Barth, M.D., and H. Roger, M.D. Translated from the French, with Notes, by P. Newbigging, M.D.—Edinb. 1842. 8vo, pp. 398.

28. An Investigation of the present unsatisfactory and defective state of Vaccination, &c. &c. By Thomas Brown.—Edinb. 1842. 8vo, pp. 139. 4s.

29. The reciprocal Influence of Body and Mind considered. By W. Newnham, Esq.—Lond. 1842. 8vo, pp. 688. 14s.

30. Transactions of the Cornwall Medical Association for the year ending the 8th February, 1842.—St. Columb. 1842. 8vo, pp. 64.

31. A Dispensatory or Commentary on the Pharmacopœias of Great Britain. By Robert Christison, M.D., F.R.S.E., &c.—Edinb. 1842. 8vo, pp. 978. 18s.

32. The Hunterian Oration, delivered at the Royal College of Surgeons in London, Feb. 16, 1842. By G. G. Babington, Surgeon to St. George's Hospital.—London, 1842. 8vo, pp. 32. 1s.

33. Lectures on the Diseases of the Urinary Organs. By Sir Benjamin C. Brodie, Bart., F.R.S., Serjeant-Surgeon to the Queen. Third Edition, with alterations and additions.—Lon. 1842. 8vo, pp. 379. 12s.

34. Principles of Human Physiology, &c. By W. B. Carpenter, M.D.—London, 1842. 8vo, pp. 680. 20s.

35. A Practical Treatise on Nervous Impediments of Speech, Stammering, &c.—Fifth Edition. By J. Poett, M.R.C.S.—Lond. 1842. 12mo, pp. 99.

36. Discourse on the enlarged and pendulous Abdomen, showing it to be a visceral affection, &c. Second Edition, with a Dissertation on Gout, &c. By R. Franklin, Esq., Surgeon.—London, 1842. 8vo, pp. 121. 5s.

37. The First and Second Reports of the Medical Missionary Society in China: with Minutes of Proceedings, Hospital Reports, &c.—Macao, 1841. 8vo, pp. 68.

FOREIGN.

1. Grundzüge einer neuen und wissenschaftlich begründeten Cranioscopie (Schädelehre). Von Dr. C. G. Carus, M.D. &c. —Stutgard, 1841. 8vo, pp. 87.

2. Nosologisch-therapeutische Untersuchungen über die brandige Zerstörung durch Behinderung der Circulation des Blutes. Von C. F. F. Hecker, M.D. Professor der Medicin, &c. zu Freiburg.—Stuttgart, 1841. 8vo, pp. 68.

3. Zwölf Briefe über das Erdleben. Von Dr. C. G. Carus, M.D. &c.—Stuttgard, 1841. 8vo, pp. 296.

4. Sulla Temperatura dell' atmosfera nell' isola di Malta. Memorie contenante i resultati delle osservazioni termometriche dall' anno 1820 all anno 1840, del Dr. Saveria Schembri.–Malta, 1841. 8vo, pp. 23.

5. Ueber das Licht und dessen Einfluss auf die natur, besonders auf den menschlichen Organismus. Ein Vortrag, gehalten von G. P. Holscher, M.D.—Hannover, 1841. 8vo, pp. 20.

6. Nouveau Manuel d'Anatomie descriptive et raisonnée, avec un Atlas de 34 Planches. Par C. Crommelinck, M.D. &c. —Bruxelles, 1841. Roy. 8vo, pp. 436.

7. Mémoire sur l' Opération du Strabismus Spasmodique. Par C. Crommelinck, M.D.—Bruges, 1841. 8vo, pp. 22.

8. De eventu Sectionis Cæsareæ. Dissertatio Inauguralis. Auctore Carolo Kayser.—Havniæ, 1841. 8vo, pp. 122.

9. De Ulcere Ventriculi perforante. Diss. Inaug. E. A. Dahlerup, auctore.—Havniæ, 1841. 8vo, pp. 77.

10. De Prolapsu funiculi Umbilicalis. Diss. Inaug. Auctore J. C. Saxtorph.—Havniæ, 1840. 8vo, pp. 68.

11. Quid faciant ætas annique tempus ad frequentiam et diuturnitatem morborum hominis adulti, disquisitio medico-statistica. Auctore E. Fenger, L.M. Univ. Havn. —Havniæ, 1840. 8vo, pp. 71.

12. Mémoire sur la revulsion morale dans le traitement de la Folie. Par F. Leuret, M.D. &c.—1841. 4to, pp. 33.

13. Om Sygpleien i Straffeanstalterne i Norge. Ved Professor Holst, M.D.—Christiania, 1841. 8vo, pp. 112.

14. Fortegnelse over de auctoriserede Læger i Norge.—Christiania, 1831. 8vo, pp. 8.

15. Mémoires, &c. Par le Docteur Jules Guerin.—Paris, 1841. 8vo.

16. Guide Pratique pour l'étude et le traitement des Maladies Syphilitiques. Par L. Ducros, M.D.—Par. 1841. 8vo, pp. 323.

17. Anatomisch-Microscopische Untersuchungen zur allgemeinen und Speciellen Pathologie. Von Gottlieb Glage, M.D., &c. Zweites Heft.—Jena, 1841. 8vo, pp. 206.

18. Femoris amputatio ex acetabulo, Caroli Dauntonis Markensis, AD. 1738-9, a Roberto Jeffrey, Chirurgo Huntspilliari, feliciter institute, ac nepote suo, Josepho Jeffrey, M.D., nunc memorata.—Coloniæ, 1841. 8vo, pp. 15.

### INDEX TO VOL. XIII.

OF THE

### BRITISH AND FOREIGN MEDICAL REVIEW.

| | PAGE |
|---|---|
| Abscess of the breast, deep . | 264 |
| of the lungs, Dr. Graves on | 557 |
| subcutaneous opening of | 241 |
| Aconitine, account of . . | 60 |
| Acoustic chair (wonderful) . | 512 |
| Albumen not antidotal of cor. sub. | 574 |
| Amputation, Mr. King on . | 172 |
| of the leg, plan for . | 565 |
| Amsterdam, Transactions of . | 503 |
| Anatomy, Treatise on . . | 518 |
| and physiology, Mr. Walker on | 205 |
| Anchylosis of knee, operation for | 12 |
| Anemia, Dr. Canstatt on . | 331 |
| Aneurism, new treatment of . | 564 |
| Angeioleucitis, M. Velpeau on . | 173 |
| Annesley, Mr. on Indian diseases | 347 |
| Anthrax, malignant . . | 43 |
| Anus, artificial, new mode of forming | 237 |
| Anus, imperforated, case of . | 508 |
| Aorta, obliteration of . . | 255 |
| wound of . . | 542 |
| Aphtha epizootica . . | 47 |
| Arntzenius on the urethra . | 312 |
| Arnott, Mr. on strictures . | ib. |
| Arrowroot, account of . . | 77 |
| Arsenic, recovery from . | 572 |
| treatises on detecting . | 183 |
| Articulations, surgery of . | 169 |
| Asphyxia from abscess . | 230 |
| Assafœtida, experiments on . | 575 |
| Auscultation, M. Fournet on . | 220 |
| Axillary artery, wound of . | 564 |
| Barry, Dr. on Fibre . . | 554 |
| Becquerel, M. on the urine . | 435 |
| Bellingham, Dr. his "Materia Med." | 521 |
| Bennet, Dr. on cod-liver oil . | 197 |
| Bethlehem Hospital, management of | 215 |
| Bladder, abscess of . . | 237 |
| puncture of . . | 241 |
| Blood-globules, production of . | 229 |
| Bloodletting in mania . | 559 |
| in pneumonia . | 402 |
| Books for review . | 288-581 |
| Bowman, Mr. on fatty liver . | 553 |
| Brady, Dr. his translation of Fournet | 220 |
| Brain, fatal wound of . . | 240 |
| pseudo-morbid appearances of | 562 |
| softening of . . | 535 |
| tubercles in . . . | 503 |

| | PAGE |
|---|---|
| Bright's disease, pathology of . | 435 |
| does it exist? . | 560 |
| Cæsarean operation, successful . | 545 |
| Cancer of womb, incipient stage of | 571 |
| Canstatt, Dr. his Therapeutics . | 327 |
| Cantharides, account of . . | 64 |
| Carbuncle, malignant, history of . | 40 |
| Carotids, ligature of . . . | 565 |
| Carpenter, Dr. his General Physiology | 160 |
| Human Physiology | 467 |
| Catherwood, Dr. on chest diseases | 220 |
| Cephalhæmatoma, Dr. Walshe on | 180 |
| Cephaloscope, the . . . | 511 |
| Charities, medical, of Ireland . | 513 |
| Chilblains, treatment of . . | 62 |
| Childmurder, case of . . . | 250 |
| Children's diseases . . | 219, 222 |
| Childs, Mr. on spinal curvature . | 221 |
| Chinese midwifery . . . | 569 |
| Chowne, Dr. his Oration . . | 219 |
| Cholera, Dr. Williams on . . | 99 |
| Chloride of lime, account of . | 62 |
| Chlorosis, Dr. Campbell on . | 332 |
| Choroiditis, cause of glaucoma . | 503 |
| Christison, Dr. his dispensatory . | 522 |
| Chrondritis, Mr. Wells on . . | 169 |
| Circulation, fœtal, Dr. Willis on . | 262 |
| Climate and malaria . . . | 257 |
| effect of on disease . | 423 |
| Club-foot, Dieffenbach on . . | 4 |
| Cod-liver oil, treatise on . . | 197 |
| Collegiate system in London . | 268 |
| Combe, Dr. on digestion . . | 219 |
| Comparative anatomy, Dr. Grant on | 217 |
| Compression, Mr. Sharp on . | 130 |
| Concussion, Mr. Sharp on . . | 124 |
| Condyloma, Mr. Johnson on . | 178 |
| Confervæ on living frogs . . | 523 |
| Congestion, Dr. Campbell on . | 333 |
| Conolly, Dr. on the treatment of the insane . . . . . | 213, 274 |
| Constitution modifying disease . | 520 |
| Contractions, operations for . | 14 |
| Copaiba, account of . . . | 67 |
| Cowpox and smallpox, non-identity of | 560 |
| Creosote, account of . . . | 68 |
| Criminals, phrenology of . . | 527 |
| Crommelinck's anatomy . . | 518 |
| Croton oil, account of . . | 69 |

PAGE
Croup, observations on . . 569
tracheotomy in . . 248
Curtis, Mr. on aural pathology . 509
Cyclopædia of surgery . . 168
Cysts, serous, treatment of . . 543
Diabetes . . . . . . 259
researches in . . 539
Diarrhœa of stokers . . . 231
Dieffenbach on tendon cutting . 1
cure of old fractures 239
Digestion, physiology of . . 219
Diseases, transmissible from animals 28
natural order of . . 202
Dislocations of femur, unreduced 420
statistics of . . 245
Dispensatory, Dr. Christison's . 522
Dissection wounds, Dr. Williams on 95
Doctors versus insurance companies 578
Danish pharmacopœia . . 85
Donellan, Dr. on erysipelas . 373
Dropsy after scarlatina . . 261
Dunglison, Dr. his new remedies 54
Dysmenorrhœa, leeches in . . 236
Dysentery, Dr. Williams on . 98
Ear, on the diseases of . 509, 517
movements of the bones in . 568
Eczema, treatment of . . . 541
Edinburgh pharmacopœia . . 79
Empyema, on the operation of . 364
Emphysema, subcutaneous . 506
Ensiform cartilage causing pyrosis 558
Entozoon in the eye of the horse 526
Epidemic disease, remote causes of 497
Epiglottis, on the functions of . 227
Ergot, production of . . . 255
Erysipelas, Treatise on . . 373
infantile . . . 376
Egot, account of . . . 73
effects of . . . 570
Evolution, spontaneous . . 568
Eye, galvanism in diseases of . 242
inflammation of . . 503
Face, division of the muscles of . 244
Farcy, history of . . . 34
Favus, seat and nature of . . 558
Ferruginous pills, preparation of . 254
Fetus, viable at six months . 255
congenital disease of . 568
Fever, Dr. Canstatt on . . 341
in India . . . 355
puerperal . . . 109
rheumatic . . . 446
use of quinine in . . 560
Fibre, Dr. Barry on . . . 554
Fistula, hepato-pulmonary . 236
Flat-foot, Dieffenbach on . . 8
Food, comparative nutriveness of 486
Formations, hetero-plastic . 339
Forry, Dr. on climate . . 423
Foundling hospitals in France 98
Fractures cured by tenotomy . 239
France, foundling hospitals of . 289
Funis, knot on . . . . 568

PAGE

Gallstones, statistics of . . 561

Galvanism in eye-diseases . . 242

Gamboge, account of . . 63

Gangrene, amputation in . . 567

Gelatine, nutritiveness of . 253, 486

Glanders, history of . . . 29

transmission of . . 235

in men . . . 538

Glaucoma, nature of . . 503

Glasgow report on food . . 486

Glottis, œdema of . . . 537

Gluten, nutritiveness of . . 490

Gonorrhœa, treatment of . . 31

contagion of . . 239

Grant, Dr. his comparative anatomy 217

Graves, Dr. on abscess of the lungs 557

Grease, the history of . . 48

Grisolle, M. on pneumonia . 382

Gravidine, a test of pregnancy . 570

Guy, Dr. on lung-tests . . 572

Hair, on the development of . 525

Hanwell, treatment of insane at . 274

Harrowgate water, artificial . 575

Head, on injuries of . . . 123

fœtal, injured in labour . 548

Health, on preserving . . . 519

of poor in Kelso . . 576

Heart, motions of . . . 256

partial hypertrophy of . 557

Heat, animal . . . . 229

as a cause of disease . . 348

Helm, Dr. on puerperal diseases . 100

Hemorrhage, from polypus . . 543

Hereditariness of insanity . . 259

Hernia, Malgaigne on . . 245

use of opium in . . 566

Hippuric acid, its tests . . 263

Hjort, Dr. on radesyge . . 461

Hodgkin, Dr. on health . . 519

Hope, Dr. on chronic pleurisy . 364

Hocken, Dr. on hysteria . . 520

the constitution . ib.

Hospitals for the insane, on . 213

foundling, history of . 289

Hydrocephalus, chronic . . 543

Hydrocyanic acid, account of . 57

Hydrophobia, Dr. Williams on . 90

history of . . 50

idiopathic . . 559

Hypertrophy, Dr. Canstatt on . 327

Hysteria, pathology of . . 520

Iceland moss, account of . . 65

Inflammation, Dr. Canstatt on . 334

Irish moss, account of . . 65

India, on the diseases of . . 347

Indigo, account of . . . 76

Insane, on the treatment of . 274

Insects, larvæ of, passed by stool 540

Insurance offices and doctors . 578

Iodine injections in cysts . . 543

Ireland, medical charities of . 513

Iron, saccharated carbonate of . 73

iodide of . . . . ib.

31

| | PAGE |
|---|---|
| Iron, proto-ioduret, formula for . | 549 |
| Irritability, muscular . . . | 226 |
| Jaw, anchylosis of . . . | 527 |
| Jacobi on hospitals for the insane | 213 |
| Johnson, Dr. on diseases of India | 347 |
| hypertrophy of the heart . . | 557 |
| Joints contracted, operations for | 20, 21 |
| effects of rest on . . | 243 |
| Kelso, health of . . . . | 576 |
| Kiwisch, Dr. on puerperal diseases | 100 |
| Kidneys in Bright's disease . | 435 |
| Kiestein, a sign of pregnancy . | 568 |
| Labour, injury of fœtus in . . | 548 |
| Leeches, mortality of . . . | 234 |
| in dysmenorrhœa . . | 236 |
| Labour, premature, mode of inducing | 249 |
| Larvæ of insects by stool . . | 540 |
| Larynx, on the nerves of . . | 226 |
| fistula of . . . | 243 |
| Laurel water, account of . . | 59 |
| Leprosy and leper hospitals, account of . . | 256-563 |
| Leprosy, Norwegian, treatise on . | 461 |
| Levin, Dr. on transmissible disease | 28 |
| Ligament, new, of the velum . | 228 |
| Lime, chloride of, account of . | 62 |
| Liver, diseases of in India . | 358 |
| fatty degeneration of . | 553 |
| Lungs, abscesses of . . | 557 |
| static tests of . . | 572 |
| Mania, on bloodletting in . | 559 |
| Martin, Mr. on Indian diseases . | 347 |
| Macleod, Dr. on rheumatism . | 445 |
| Madness, a poem on . . | 412 |
| Mathematics and medicine . | 257 |
| Man, popular lectures on . | 222 |
| natural history of . . | 521 |
| Materia medica, works on . | 54-521 |
| Medicine, Dr. Williams' elements of | 89 |
| Mercury, iodide of . . | 74-5 |
| Metallic tinkling, cause of . | 536 |
| Milk, account of . . | 78 |
| Miners and sappers, diseases of . | 532 |
| Monstrosity, remarkable . | 527 |
| Montgomery, Dr. on cancer . | 571 |
| Mortality of London . | 267-577 |
| Muscles and tendons, division of . | 1 |
| Muscular retraction, syphilitic . | 543 |
| Myelitis infantum . . | 248 |
| Myopia cured by operation . | 238 |
| Nervous action, M. Longet on . | 226 |
| Neuralgia, Valleix on . . | 136 |
| trifacial . . | 143 |
| cervico-occipital . | 149 |
| cervico-brachial . | 149 |
| dorso-intercostal . | 150 |
| lumbo-abdominal . | 152 |
| crural . . | 153 |
| femoro-popliteal . | 154 |
| treatment of . | 156 |
| Neuroses, Dr. Canstatt on the . | 343 |

| | PAGE |
|---|---|
| Nitrate of silver, account of . | 61 |
| Nose, cure of obliquity of . | 242 |
| Nunneley, Mr. on erysipelas . | 373 |
| Œdema of the glottis . | 537 |
| Oleum jecoris aselli . . | 197 |
| Oliver, Dr. his physiology . | 222 |
| Ophthalmia and other diseases . | 522 |
| Orfila, M. on arsenic . | 183 |
| Os uteri, closure of . . | 566 |
| Ovary, extirpation of . . | 547 |
| Polypus of uterus, treatment of . | 543 |
| Porrigo, treatment of . . | 97 |
| Portio dura, paralysis of . | 553 |
| Pregnancy, ventro-uterine . | 507 |
| Pregnancy, extra-uterine . | 545 |
| tests of . | 568-570 |
| Presentations in labour . . | 570 |
| Prichard, Dr. his history of man . | 521 |
| Prolapsus of the uterus . | 563 |
| Puerperal diseases, on . | 100 |
| Pulmonary diseases, of climate on | 423 |
| Pulse of infants . . | 547 |
| Pustule, malignant . . | 44 |
| Pyrosis from pressure . . | 558 |
| Phillips, M. on the division of muscles | 1 |
| Mr. his London Pharmacopœia | 54 |
| Phlebitis, uterine . . | 104 |
| Phrenology in a penitentiary . | 527 |
| Physometra, case of . . | 246 |
| Physiology, general, Dr. Carpenter on | 160 |
| human, Dr. Carpenter on | 467 |
| Dr. Oliver on . | 221 |
| Pilcher, Mr. on diseases of the ear | 517 |
| Plague, Dr. Williams on . | 94 |
| not contagious . | 258 |
| Plaster, Liston's . . | 265 |
| Pleurisy, chronic, treatment of . | 364 |
| Pneumonia, treatise on . | 382 |
| signs and symptoms of | 393 |
| Poisoning from a minute dose of laudanum . . . | 574 |
| Paralysis of the face . | 235-241 |
| from visceral disease . | 562 |
| Paraphymosis . . | 565 |
| Parasitic formations . . | 232 |
| Parisian Report on Food . | 486 |
| Parotid, hypertrophy of . | 567 |
| Parkin, Dr. on epidemic disease | 497 |
| Pathology founded on physiology | 205 |
| and therapeutics, Canstatt's | 327 |
| Pemphigus, contagion of . | 533 |
| Pereira, Dr. his Materia Medica . | 54 |
| Perforation of the stomach | 516-563 |
| Pharmaceutical Society . | 211 |
| Pharmacopœias, various . | 54 |
| Pharmacy, works on . . | ib. |
| Quinine in fevers . . | 560 |
| Ranunculus, effects of . | 232 |
| Rees, Mr. on children's diseases . | 219 |
| Reform, medical . . | 580 |
| Respiration, on the movements of | 223 |
| Revaccination in Prussia . | 550 |

| | PAGE |
|---|---|
| Revaccination in Hanover . | 551 |
| Rheumatism of the skin . | 423 |
| effect of climate in . | 423 |
| Dr. Macleod on . | 445 |
| deformity from . | 566 |
| Radesyge . . . | 461 |
| Rayer, M. on glanders . . | 28 |
| Russian Pharmacopœia . . | 84 |
| Saliva, sulpho-cyanogen in . | 260 |
| Sappers and miners, diseases of . | 532 |
| Scabies ferina . . | 49 |
| Scarlatina, state of urine in . | 537 |
| Scrofulosis and tuberculosis . | 339 |
| Scurvy, nature of . . | 492 |
| Secretions, anomalies of . | 338 |
| Sedillot, M. on empyema . | 364 |
| Setons, use of . . . | 261 |
| Sharp, Mr. on injuries of the head | 123 |
| Skin, stony concretions in . | 240 |
| rheumatism of . . | 234 |
| Softening of the brain . | 535 |
| Spasm, muscular, cured by operation | 22 |
| Spinal curvature, dissection of . | 564 |
| operation for . | 566 |
| Spine, lateral curvature of . | 221 |
| rupture of . . | 542 |
| Spleen, diseases of in India . | 362 |
| Squinting cured without operation | 239 |
| Stevens, Mr. his new nosology . | 202 |
| Stewart, Dr. on children's diseases | 222 |
| Stomach, perforation of . . | 516 |
| Streeten, Dr. his Address . . | 520 |
| Strictures, treatises on . . | 312 |
| Subjectivity of disease . . | 260 |
| Submaxillary gland, extirpation of | 238 |
| Sugar, nutriveness of . . . | 490 |
| Suicide, statistics of . . . | 552 |
| Surgery, Cyclopædia of . . | 168 |
| Simpson, Dr. on leprosy . . | 563 |
| Sweating sickness in India . . | 355 |
| France . | 534 |
| Syrian medical-aid association . | 579 |
| Tartar emetic in pneumonia . | 409 |
| Tartarised antimony, account of . | 60 |
| Tendons and muscles, division of | 1 |
| Tenotomy, subcutaneous . . | ib. |
| Thæria or radesyge . . . | 461 |
| Tracheotomy in croup . . | 248 |
| successful case of . | 565 |
| Trismus, neonatorum cause of . | 528 |
| epidemic . | 249 |
| Tuke, Mr. on insane hospitals . | 213 |
| Tubercles of the brain . . | 503 |
| Tuberculosis and scrofulosis . | 339 |
| Tinea, new etiology of . . | 233 |
| mode of treating . | 244 |
| Typhus apoplectic epidemic . | 529 |
| vesical epidemic . . | 541 |
| Ulcers between the toes . . | 562 |
| Urea in the blood . . . | 228 |
| urine, state of . . | 253 |
| Urethra, stricture of . . . | 312 |
| anatomy of . . . | ib. |
| Urine, albuminous . . . | 262 |
| state of in scarlatina . | 537 |
| Uterus and vagina, double . | 228 |
| inversion of . . . | 548 |
| prolapsus of . . . | 549 |
| Vaccine virus . . . . | 559 |
| Vagina, double . . . . | 228 |
| laceration of . . . | 568 |
| Valleix, M. on neuralgia . . | 136 |
| Velpeau, M. on angeioleucitis . | 173 |
| Vertebræ, dislocation of . . | 266 |
| Volcanoes, alleged cause of epidemics | 497 |
| Vrolik, M. on dislocation . | 420 |
| Vulva, inflammation of follicles of | 546 |
| Wade, Mr. on stricture . . | 312 |
| Walker, Mr. on anatomy and physiology . . | 205 |
| Walshe, Dr. on empyema . | 364 |
| White, Mr. his lectures on man . | 222 |
| Williams, Dr. his elements of medicine | 90 |
| Worm, intestinal, rare . . | 532 |
| Wylie, Sir J. his pharmacopœia . | 54 |
| Yearsley, Mr. on the ear . . | 509 |

END OF VOL. XIII.

PRINTED BY C. ADLARD, BARTHOLOMEW CLOSE.

